# Evaluation Method of the Driving Workload in the Horizontal Curve Section Based on the Human Model of Information Processing

**DOI:** 10.3390/ijerph19127063

**Published:** 2022-06-09

**Authors:** Huan Liu, Jinliang Xu, Xiaodong Zhang, Chao Gao, Rishuang Sun

**Affiliations:** 1 School of Highway, Chang’an University, Xi’an 710054, China; gaochao@chd.edu.cn; 2 Journal Center, Chang’an University, Xi’an 710061, China; 2017021039@chd.edu.cn; 3 Shandong Provincia Conmmunications Planing and Design Institute Group Co., Ltd., Jinan 250101, China; handsomepb@139.com

**Keywords:** traffic safety, driving workload, human model of information processing, horizontal curve section, ECG indexes, NASA-TLX scale

## Abstract

The aim of this study was to quantify the effect of radius over horizontal curve sections on driving workload (*DW*). Twenty-five participants participated in the driving simulation experiments and completed five driving scenes. The NASA-TLX scale was used to measure the mental demand, physical demand, and temporal demand in various scenes, which were applied to assess subjective workload (*SW*). Objective workload (*OW*) assessment methods were divided into three types, in which the eye tracker was used to measure the blink frequency and pupil diameter, and the electrocardiograph (ECG) was used to measure the heart rate and the heart rate variability. Additionally, the simulator was used to measure the lateral position and the steering wheel angle. The results indicate that radius is negatively correlated with *DW* and *SW*, and the *SW* in a radius of 300 m is approximately twice that in a radius of 550 m. Compared with the ECG, the explanatory power of the *OW* can be increased to 0.974 by combining eye-movement, ECG, and driving performance. Moreover, the main source of the *DW* is the maneuver stage, which accounts for more than 50%. When the radius is over 550 m, the *DW* shows few differences in the maneuver stage. These findings may provide new avenues of research to harness the role of *DW*s in optimizing traffic safety.

## 1. Introduction

Sharp curve sections are accident-prone [1], and more than 60% of traffic accidents are due to an improper *DW* caused by horizontal curve sections [2,3]. Based on the Yerkes–Dodson law [4], Reid proposed that there is an optimal workload level in any driving task and that a workload that is too high or too low leads to poor performance [5]. When a driver encounters a sharp curve, the driver is forced to bear a high workload and is prone to make emergency steering and braking errors. Therefore, verifying the influence of the radius on the *DW* is necessary, which helps to compensate for the lack of human factor consideration in circular curve design [6].

The methods of measuring *DW* mainly include scale measurements, physiological measurements, and performance measurements. Lateral position (*LP*) is a key indicator of driving behavior. Fu [7] found that the mutation of spatial curvature is positively correlated with the maximum *LP*. Because of the difficulty of selecting experimental sections, the results made it impossible to ignore the influence of gradient change. A simulation study of the radius-only variable by Lin [8] reported that the radius potentially represented negative safety implications for driving performance, and the influence was that the standard deviation of the *LP* increased by the radius reduction. Portera and Xu observed that the lateral motion also worked worse along curved sections [9,10]. However, Wu [11] demonstrated an inconspicuous correlation between the radius and the standard deviation of the *LP* because a lower speed limit in the experiments stabilized the steering control. Thus, the driving behavior was significantly dependent on the speed, and other studies on workload have shown that the radius has a similar negative influence on the steering wheel angle (*SWA*) [12] and lateral acceleration [13,14]. In particular, Peter and Easa [15,16] indicated an essential point as their study reported that the main disadvantage of the horizontal curve section is the disorientation caused by increasing centripetal force, and the disorientation results in a significant increase in the standard deviation of the *LP*, which leads to accident rates of more than 40% [17].

A study reported that the change in driving behavior may not easily explain the mental workload of driving [18]. Then, the scale measurement attempted to detect the mental workload. Commonly used scale measurement methods include the National Aeronautics and Space Administration Task Load Index (NASA-TLX) [19], Subjective Workload Assessment Technique [20], and Workload Profile [21]. The NASA-TLX is considered to be one of the most systematic assessments of mental workload [22] and can measure *SW* in various aspects, such as mental demand, physical demand, temporal demand, performance, frustration level, and effort. Furthermore, the NASA-TLX has been shown to lead to success in driving. Xie [23] indicated that drivers who experienced a sharp curve scored higher on the NASA-TLX. However, the participants were of a similar age. Therefore, participants aged from 21 to 24 and from 29 to 33 were invited to participate in field experiments at a uniform speed. An interesting result is that the *SW* is higher for young drivers in general, especially on curved sections [24].

The main physiological indexes used to measure driving workload include eye-movement [25,26,27,28], electroencephalogram [29,30], ECG [31], and electromyographic signals [18]. Not every index is sensitive to changes in the radius, with evidence suggesting that eye-movement is significantly affected by radius [32]. The smaller the radius displayed to a driver at a given time, the larger the pupil diameter (*PD*) for the driving process [33,34]. At the same time, the radius also influenced the characteristics of ECG, such as heart rate (*HR*) and heart rate variability (HRV) [35]. The horizontal curve section presents more complex curvature variations, which require drivers to leverage more attention resources for lane-keeping. This undoubtedly increased the changes in *HR* and HRV. Such a complex curve scene will lead to a higher workload and lower blinking frequency, as shown in field driving conditions [36]. However, this research is limited in that the results failed to quantize the relationship between *DW* and radius. Furthermore, Zheng [37] developed a quantitative model between the growth of *HR* (*GRHR*), speed, and the radius from 200 m to 2000 m in the field of driving. The model showed that a radius of 500 m is the threshold for a significant negative effect on drivers’ physiology. It is difficult to collect complete and continuous data on physiological measurements in both field driving and simulation driving [38].

While these studies have revealed the variation rules of driving performance, subjective score, and physiological indexes in the horizontal curve sections, few researchers have attempted to accurately determine *DW*. Waard [39] analyzed a combination of the above indexes using a driving simulator, and the results primarily demonstrated that the sensitivities of each index varied with task difficulty. Therefore, a single-index approach to assessing *DW* is incomplete and inaccurate.

Workload is typically defined as the difference between the perceived effort and actual effort, including subjective and objective aspects [40]. The absence of any aspect can lead to a lack of credibility in workload measurement [41]. Accordingly, Hancock [42] claimed that workload is the product of an operator mobilizing attention resources to meet task demands. When the task demands exceed the available resources, operators need to adjust their strategies or the performance will inevitably deteriorate. However, resources are not fixed and depend on individual ability. In addition, the operator will make a subjective assessment of the resources needed for a task [43], that is, *SW*.

With the contemporary rapid development of human factor research, attention resources are multidimensional because they differ in the processes of information acquisition, decision-making, and response [44]. Similarly, workload has multidimensional properties. Based on the above opinions, many studies have innovatively combined the human model of information processing (HMP) to realize workload models related to human–computer interactions, such as aircraft [45] and roads [46]. For example, the queueing network model of *DW* [47] proposed that the quantitative approach is more accurate when the *DW* is concentrated in the perceptual, cognitive, or maneuver stage. A key limitation of this study is that the results were obtained by the NASA-TLX, which was used to assess *SW* rather than *DW*. Therefore, how to significantly extend the HMP to cover *DW* requires further research.

In summary, from the theory of the HMP, this research intends to evaluate the driving workload over curve sections by following key steps. (1) The NASA-TLX scale is used to measure the mental demand, physical demand, and temporal demand in various scenes to assess the *SW*. (2) The *OW* assessment methods are divided into three types, in which an eye tracker is used to measure the blink frequency (*BF*) and *PD*; an ECG is used to measure the *HR* and root mean square of successive differences between adjacent RR intervals (*RMSSD*); and a simulator is used to measure the *LP* and *SWA*. (3) By arranging the standard value, which is the mean index of the straight-line section, the *SW* and *OW* are integrated. (4) The weight of driving workload from each stage (SDW) (the visual–perceptual, cognition, and maneuver stages) was determined by classification algorithms. A machine-learning-based workload modeling approach was proposed by dynamically monitoring drivers’ psychological and physiological indicators. Such an approach may improve the sustainability and resilience of drivers and intelligent transportation systems. It may also help to build interdisciplinary intelligence systems for digital health.

The rest of this paper is organized as follows. First, the *DW* definition and modeling method, including the driving simulation experiments and data processing, are presented in Section 2. Then, we measure the *SW*, *OW*, SDW, and *DW* at each stage in Section 3. The research is discussed in Section 4 and summarized in Section 5.

## 2. Materials and Methods

### 2.1. Experimental Design

The experiments were designed on a driving simulator. Driving simulators are safe and effective platforms that combine an ECG module, eye-movement module, and behavior output [48]. Current evidence suggests that simulation driving data differ from field driving data, but the regularity may be similar [49,50]. In other words, the data collected by a driving simulator lack absolute validity but have relative validity. Therefore, this research selected the radius as a variable to verify the driving simulation validity; the radius was taken as variable for the selection of field test roads.

### 2.2. Experimental Scenes

This study determined the radius as an independent variable to improve subjects’ capture of curve changes. Fitzpatrick [51] indicated that the effect on driving behavior, such as speed selection, is less when the radius is greater than 550 m. To exclude the influence of circular curve length and superelevation on the experimental results, the radius (300 m, 550 m) was taken as the research object. The circular curve length was set to approximately 260–280 m to meet the requirement of 3 s of travel, and the maximum superelevation was set to 8%.

For the distance before entering a curve, drivers can perceive the curve visually and adjust their driving behavior. A dynamic vision study [52] found that the driver’s gaze distance is approximately 377 m, and the deepest vision distance is 500 m when the speed is 80 km/h. Therefore, this paper selects 500 m before the clothoid to the end of the clothoid as the horizontal curve section. In addition, the research set five scenarios by selecting a flat terrain area in eastern China on UC-win/Road modeling software. A relevant study reported that speed and heart rate growth rate were highly correlated [53]. The section type is a two-way four-lane highway with a design speed of 80 km/h and a speed limit of 80 km/h. The specific parameters of the driving simulation model are shown in Table 1.

**Figure 1:**
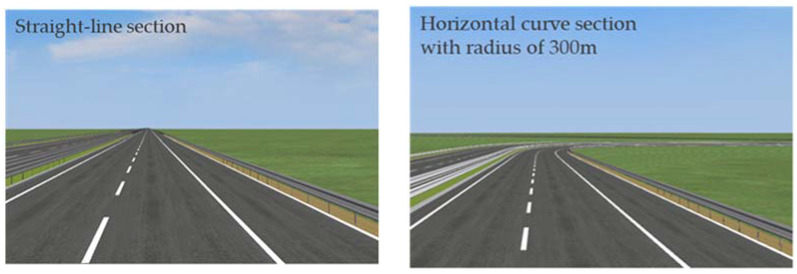
The experimental scenes.

To ensure the reliability of the driving simulation, the experimental scene has no input of traffic flow other than the test vehicle. Speed limit signs were set on the right side of the road, and the location was 200 m away from the beginning of the section. The layout and setting of road signs and markings were set according to the Specification for Layout of Highway Traffic Signs and Markings (2009) in China. The weather for all scenarios was clear and well lit, as shown in Figure 1. With a similar driving scene, a horizontal curve section with a radius of 500 m in central China was selected for the field experiment to verify the relative validity of the simulation results.

A field investigation was carried out on Xian-Xun freeway. We selected one curve section with radius of 500 m. The simulated road was established according to real road parameters, and the side environment of the simulation road is consistent with the actual road. Meanwhile, the experiment time was taken during the week between 9:00 AM and 11:00 AM to ensure that the test vehicle was not affected by traffic flow.

### 2.3. Participants

Twenty-five participants (12 males and 13 females), ranging in age from 30 to 55 years (mean = 42; SD = 6.8), were recruited for this research. All were social workers with a high educational degree and good physical health. These participants had valid driving experience of 7 to 16 years (mean = 12.4; SD = 8.2), with an average of 20,000 km or more driven per year. According to *the Logarithmic visual acuity chart* (4.0–5.2) implemented in 2012 in China, the corrected visual acuity of the participants was 5.0 or high. Prior to the experiment, none of the participants had experienced a driving simulator and Xian-Xun freeway, and all participants signed an informed consent form.

### 2.4. Experimental Equipment

**Figure 2:**
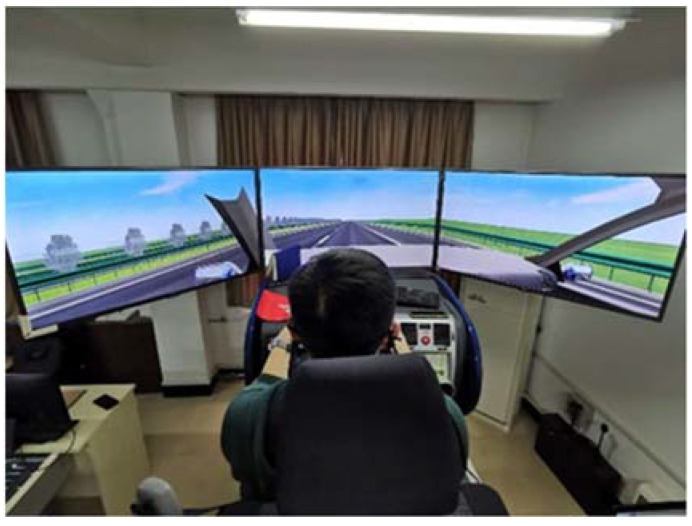
Driving simulation system.

The driving simulation system enables six degrees of freedom of motion in space (the six degrees of freedom are vertical motion, lateral motion, longitudinal motion, tilting, rolling, and swinging, that is, translation in X, Y, and Z direction and rotation around X, Y, and Z direction), which maximally simulates the field of driving scene “vehicle–road–driver” and can quickly collect vehicle motion information, including speed, acceleration, and lateral position. The driving simulator consists of three independent identical screens (screen size: 961 mm × 567 mm × 55 mm) and provides the participants with a 130-degree horizontal view and a 40-degree vertical view, as shown in Figure 2.

A Passat was selected as the field test car, and vehicle parameters were set in the UC-win/Road software in accordance with the Passat applied in the field test.

The MP160 multiple electroconductive physiological recorder was used for real-time acquisition of the participants’ ECGs. The recorder was used in conjunction with AcqKnowledge 5.0 to analyze the driver’s ECG. The sampling frequency was set to 1000 HZ.

The SMI eye tracker was used to capture the participants’ eye-movements in combination with the Iview to collect high-precision data, such as *PD*. The sampling frequency was 250 HZ. Begaze 3.7 was used to analyze the eye-movement indexes.

The WTRTK-4G High-precision positioning sensors were used to obtain the *LP* in real time and save it through the matched software CP210X tools in the computer.

The HWT101DT attitude angle sensor was used for real-time acquisition of the vehicles’ *SWA*. The data were saved by the supporting software MiniIMU tools in the computer.

### 2.5. Experimental Procedure

The simulation experiment was divided into 3 phases, including the preparation phase, pre-experimental phase, and experimental phase. In the preparation phase, the researchers calibrated the experimental equipment, the simulation models were imported and calibrated into the 5 experimental scenes, and the participant’s heart rate was measured at rest. In the pre-experimental phase, the participants traveled for 5 min on a nonexperimental scene to familiarize themselves with the driving simulation. Before the experimental phase, the participants were informed of test precautions and driving requirements, including maintaining a lane of driving without answering the phone, chatting, and other behaviors unrelated to driving tasks. In the experimental phase, all participants were required to drive five scenes for approximately 20 min. The ECG was equipped with electrode sheets, and the SMI was equipped with eyeglasses. The participants were allowed to stop the experiment when they felt uncomfortable. Between each experimental scene, the participant had a 10-min rest period without ECG or SMI to eliminate discomfort. At the same time, the participants were asked to complete the NASA-TLX scale and ensure that their heart rates returned to baseline. Then, the participants were assisted in wearing and commissioning the experimental equipment for 3 min.

This process was repeated for the other four experimental scenes. Once a participant had completed all experimental scenes, the next participant was prepared to enter the experimental area. This process was continued until all participants had completed all experimental tasks. The experimental procedures of the field test were the same as those of the simulation test.

### 2.6. Driving Workload Indexes and Evaluation Method

#### 2.6.1. Proposing Definition of Driving Workload

The controversial point of these studies is that the definition of workload is hard to agree on, and different definitions will result in different indicators. According to the driving characteristics, the HMP was applied in this paper. The HMP has three stages: perceptual, cognitive, and maneuver stages, and each stage was affected by attention resources. However, the resources in each task were limited due to the task demand. If one stage took up more resources, others took less [54]. Since *DW* reflects the perception and effort of information processing throughout each stage, *DW* may be simplified to the difference between task demands and attention resources.

The definition of *DW* in this study is the difference between task demands (*SW*) and attention resources (*OW*) caused by visual–perceptual (VP), cognition (C), and the maneuver stage (M). *SW* is composed of three aspects: VP demands, C demands, and M demands. Similarly, *OW* is composed of VP resources, C resources, and M resources. The *DW* based on the definition can be quantitative, as shown in (1).

(1) $$DW_{i}=SW_{i}-OW_{i}$$

where i represents the processing of VP, C, and M. $DW_{i}$ is the SDW of each stage, and the dimension is 1. The scores of $SW_{i}$ and $OW_{i}$ represent the *SW* and *OW* of each stage, and their dimension is 1.

#### 2.6.2. Subjective Workload Indexes

Three indicators were used to evaluate *SW*, including mental demand, physical demand, and temporal demand, all of which were collected by the NASA-TLX scale. One report demonstrated that the NASA-TLX shows a higher sensitivity in assessing *SW* [55]. The scale consists of six parameters of demand, which are composed of mental demand (MD), physical demand (PD), temporal demand (TD), performance, frustration level, and effort. The score of any aspect ranges from 0 to 100. Except for the performance, the greater the score of other parameters, the greater the demand. The scores of MD, TD, and PD represent the VP demands, C demands, and M demands, respectively.

#### 2.6.3. Objective Workload Indexes

*OW* is the attention resources in the driving process, assessed by eye-movement, ECG, and performance. This study attempted to apply physiological measurements and performance measurements in assessing *OW*.

(1) Physiological indicators

Based on the correlation between mental workload and ECG, including *HR* and *RMSSD* [12,56], the drivers had to perform mental calculations during the cognitive stage. Therefore, *HR* and *RMSSD* are devoted to assessing the *OW* of the cognitive stage. Research on driving has indicated that *HR* varies greatly with individuals and relates to age, gender, and health [57]. To reveal the effect on individuals, *GRHR* was replaced to characterize the effects of driving tasks, as illustrated in Equation (2).

(2) $$GRHR=\left(HR-HR_{rest}\right)/HR_{rest}*100$$

where *GRHR* is the percentage of *HR* increment relative to the resting state, %. $HR_{rest}$ is *HR* in the rest state.

*RMSSD* indicates the root mean square of successive differences between adjacent RR intervals, which is a common time-domain index of HRV, as shown in Equation (3).

(3) $$RMSSD=\sqrt{\frac{\sum_{i=1}^{i=n-1}D_{i}^{2}}{n-1}}$$

where $D_{i}$ is the length of *RR_i_* and *RR_i_*_+1_ between two adjacent heartbeats. $D_{i}=RR_{i}-RR_{i+1}$. indicates the number of normal heartbeats.

Eye-movement is highly related to the visual perception of drivers. Related research has shown that *BF* and *PD* reflect the visual perception of a horizontal curve section [27]. Similar to the *GRHR*, the same quantitative method was applied to *PD* and *BF*. They are defined as the pupil diameter growth rate (*GRPD*) and the blink frequency growth rate (*GRBF*) as follows:

(4) $$GRPD=\left(PD-PD_{rest}\right)/PD_{rest}*100$$

(5) $$GRBF=\left(BF-BF_{rest}\right)/BF_{rest}*100$$

where *PD* represents the pupil diameter in mm. *BF* is the blink frequency per minute. $PD_{rest}$ and $BF_{rest}$ indicate the pupil diameter and blink frequency in the resting state.

(2) Driving performance

Current research has reported that *LP* and *SWA* are directly related to driving performance in horizontal curve sections, which are commonly used to reflect the maneuver workload on drivers [58,59]. Thus, two indicators are used to evaluate the maneuver resources of *OW*, including *SWA* and *LP*, as follows.

(6) $$SWA=Deg*SWA_{max}/2$$

(7) $$LP=\left|D_{RB}-D_{C-RB}\right|$$

where $SWA_{max}$ represents the maximum *SWA*: 900°. $D_{RB}$ is the lateral distance of the vehicle to the right boundary of the road derived from the driving simulator; $D_{C-RB}$ is the distance between the center and right boundary of the driving lane, and the value is set to 3.9367 m.

#### 2.6.4. Establishing Workflow of Driving Workload

**Figure 3:**
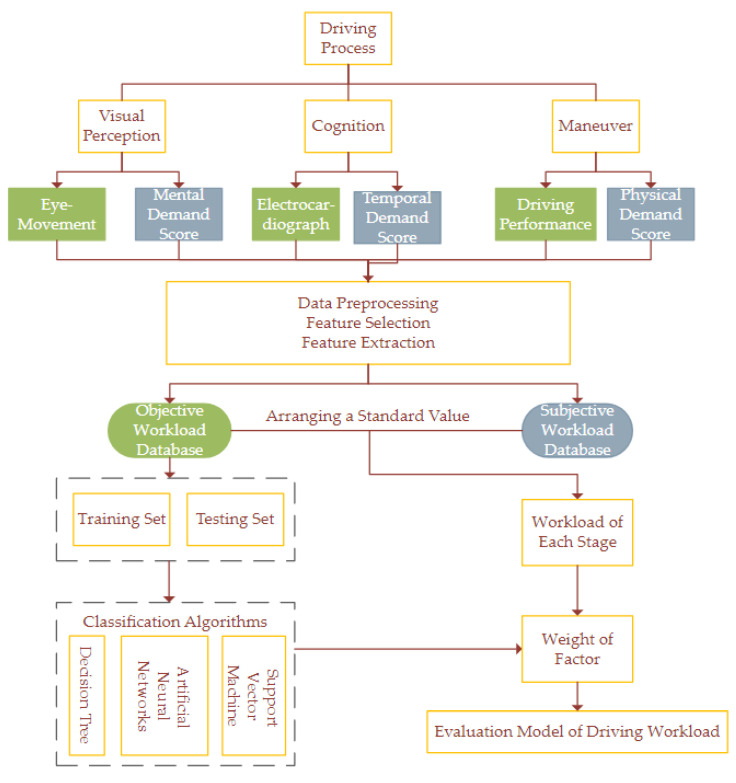
Modeling framework for the evaluation method of the driving workload.

According to the complexity of the experimental scene, the quantitative model of *DW* assumed a significant difference in the *DW* level of different experimental scenes. Additionally, it is necessary to prove the same difference in eye-movement, ECG, and driving performance because of the large dimension difference in indicators and the limited attention resources. The key methods of *DW* modeling are the correlation strategy of *SW* and *OW*, as well as the strategy of effectively quantifying SDW distribution problems in visual–perceptual, cognitive, and maneuver stages. The specific modeling framework is shown in Figure 3. Modeling framework for the evaluation method of the driving workload.

Before quantitating the driving workload, the data preprocessing was used for feature selection and feature extraction. In this study, it was conducted as follows.

(1) Pearson correlation analysis

Pearson correlation analysis was used in this study because the data redundancy caused by strong correlation features can reduce model accuracy. The purpose is to analyze the correlation between the *OW* indicators to screen *DW* evaluation indicators.

(2) the analysis of variance (ANOVA)

ANOVA was used to determine the magnitude of the influence of a variable on the results. The Mann–Whitney (M–W) test and Kolmogorov–Smirnov (K–S) test were used in this study to analyze the differences in distribution between simulator data and field data. To verify the validity of the sample, the Friedman test was used to analyze the subjective and objective data variability between individuals. The joint hypotheses F test and the Kruskal–Wallis H test were applied to analyze the effect of radius on *GRHR*, *GRPD*, and *LP*.

Since then, the mean values of MD, TD, and PD were devoted to quantitate *SW* of each stage, and the standard deviations of *GRPD*, *GRHR*, and *LP* were devoted to calculate *OW* of each stage. By arranging the standard value, which is the mean index of the straight-line section, the *SW* and *OW* were integrated to evaluate SDW. Finally, the classification algorithms were used to determine the weight of each stage of *DW* in this study. To avoid errors by absolute numerical difference, all data were standardized by the extreme value processing method.

Classification algorithms, including regression trees (RTs), Bayesian networks, logistic regression, random forests, support vector machines (SVMs), and artificial neural networks (ANNs), are often used to analyze the sensitivity of indicators to workload [60]. The data in this paper present the characteristics of small volume, with more dimensions and noise interference. SVMs can effectively solve the problem of small samples with high dimensionality and nonlinear characteristics. ANNs are not easily affected by noise and robustness. RTs have the characteristics of strong stability and anti-overfitting ability. This study adopted the above three classification algorithms to analyze the *OW* indexes.

## 3. Results

### 3.1. Analysis of Driving Workload Evaluation Index

#### 3.1.1. Selection of the Driving Workload Evaluation Index

Six indicators were used to evaluate *OW*: two items were related to ECG (*GRHR*, *RMSSD*), two items were related to eye-movement (*GRPD*, *GRBF*), and two items were related to driving performance (*SWA*, *LP*). Pearson correlation analysis was used to determine the relationships between the indicators. IBM SPSS 26 software was used for data analysis, as shown in Table 2.

In terms of the driving performance, the differences between the *SWA* and *LP* in every scene were statistically significant. The *SWA* was significantly correlated with the *GRHR* (*p* = −0.372) and *RMSSD* (*p* = −0.596). The *LP* can reflect the driving stability more intuitively. Thus, the *LP* was devoted to assessing the *OW* of the maneuver stage. For the ECG, the *RMSSD* showed a significant correlation with *GRHR* and was similar to *LP* (*p* = −0.554) and *SWA* (*p* = −0.596). Compared to the *RMSSD*, the *GRHR* can reflect more characteristics of ECG. Therefore, the *GRHR* was used to evaluate the *OW* of the cognitive stage. For eye-movement, the *GRBF* was independent of *GRPD* but was highly correlated with *LP* (*p* = −0.415) and *RMSSD* (*p* = 0.368). In the same way, the *GRPD* was applied to assess the *OW* of the visual–perceptual stage.

#### 3.1.2. Driving Simulation Validation

The purpose of this section is to judge whether the simulation results have relative validity. This study took the horizontal curve section with a radius of 500 m as an example. The M–W test and K–S test were used to verify the distribution between the field data and simulation data, including the NASA-TLX scores, *LP*, *GRHR*, and *GRPD*. A basic assumption in this study was that the differences between the two samples were not significant. The results of pairwise comparisons are shown in Table 3.

As shown in Table 3, the K–S and M–W testing results were absolutely effective on the five characteristic points of the curve sections, all of which were consistent with the assumption. Therefore, the results showed that the sample had a high consistency between the driving simulator and the field scene. Applying driving simulation to study the influence of the radius on the *DW* is reliable and reasonable.

Otherwise, the Friedman test was used to analyze the above indicators to exclude the influence of individuals on the *DW* model. The results showed that the data of each group followed a normal distribution (*p* ≈ 1).

### 3.2. Quantification of Workload in Each Stage

#### 3.2.1. Drivers’ Subjective Workload Scores

To assess the driver’s *SW*, the MD, TD, and PD results of the 25 participants were analyzed in this study, the Cronbach’s alpha test, KMO test, and Bartlett test showed that the data of each radius were significantly different (α = 0.952 > 0.7; KMO = 0.894 > 0.6, *p* < 0.05). Therefore, this research identified the mean values of the MD, TD, and PD as the *SW* of the perceptual, cognitive, and maneuver stages ($SW_{i}$), respectively. As shown in Table 4, the lower *SW* is represented by the lower score.

As observed from the *SW* scores, when the radius increases, the *SW* score reduces synchronously in each stage, and the lower *SW* scores are mainly concentrated on the straight-line section.

#### 3.2.2. Drivers’ Objective Workload Scores

Nonparametric tests were used for the mean values of *PD*, *HR*, and *LP*, which indicated that the *LP* and *PD* complied with a normal distribution. Testing by joint hypotheses F methods, the *LP* showed a significant difference, where F (−10.31, 3.80) = 5.172 and *p* < 0.05. The interaction between the *LP* and the radius was statistically significant, where F (2.90, 2.94) = 16.339 and *p* < 0.05. The cases that did not meet the normal distribution were analyzed by the Kruskal–Wallis H test. The results show that the *HR* complies with the same difference (H = 79.313, *p* < 0.05) of different radii.

Assessing the *OW* is another key step in quantifying the *DW*. The linear influence factor ($LIF_{ij}$, where j represents the radius of 300 m, 400 m, 500 m, 500 m, 550 m, and ∞) was devoted to quantitatively measure the *OW*, which indicated the effect of the radius on the *OW*. The factor was defined with the standard deviation of the *GRPD*, *GRHR*, and *LP*, as illustrated in Table 5.

As shown in Table 5, similar to the *SW*, the straight line has the least influence on the *OW*. A key strategy in *DW* modeling is relating *OW* to *SW*. Therefore, the *SW* of the straight-line section in this study was defined as a standard value. The *OW* quantitative model was proposed in Equation (8).

(8) $$OW_{i}=\frac{LIF_{ij}}{LIF_{i_{standard}}}SW_{i_{standard}}$$

Combining Equation (8) and the linear influence factor results, the results of the *OW* are shown in Table 6. A lower score represents less effort put into driving.

#### 3.2.3. Workload of Each Stage over Horizontal Curve Sections

According to the definition of the *DW* in Section 2.6.1 (Equation (1)), the SDWs of different stages in the horizontal curve sections are summarized in Table 7. When the SDW score is lower, the difference between the *SW* and *OW* is smaller. Naturally, drivers are in a more relaxed driving experience. Meantime, the *DW* of standard section (straight-line section) is defined as 0.

### 3.3. Driving Workload Evaluation Model on Horizontal Curve Sections

To determine the weight of each stage of *DW* in this study, 129,575 *OW* samples extracted in the repeated experiment of Section 2.4 were analyzed. Eighty percent of the items were randomly selected as the training set, and the others were selected as the testing set. The ANNs adopted the Levenberg–Marquardt function, and the number of hidden layer neurons was two. The sigmoid kernel function was used in the SVM. The Gini function was used to optimize the RTs with the minimum samples contained in the leaf node. The results of the classification algorithm are shown in Table 8.

As shown in Table 8, the SVM achieved higher classification accuracy in the training set based on the *GRHR*, *GRPD*, or *LP* alone, where the accuracy was 0.719, 0.468, and 0.856, respectively. Similar results were obtained in the testing set. While the input indexes were arranged as a pairwise combination in the *GRHR*, *GRPD*, and *LP*, the accuracy of the RTs showed the highest result, where the accuracy was 0.881, 0.912, and 0.946, respectively. In particular, the RTs achieved higher accuracy when the *GRHR*, *GRPD*, and *LP* were combined, where the accuracy was 0.974 in the training set and 0.948 in the testing set. Compared to the input of a single indicator or a pairwise combination, the accuracy was increased by 0.761 and 0.093, respectively. The results demonstrate that the evaluation method for the *DW* based on the multifeature combination, including the ECG, eye-movement, and driving performance, significantly outperforms the method based on the above indicators alone. In addition, compared to the ANNs and SVM, the RTs achieved higher average accuracy based on their strong stability and anti-overfitting ability. Thus, the results of the RTs (Figure 3) were devoted to evaluating the sensitivity of the SDW for the driving workload.

**Figure 4:**
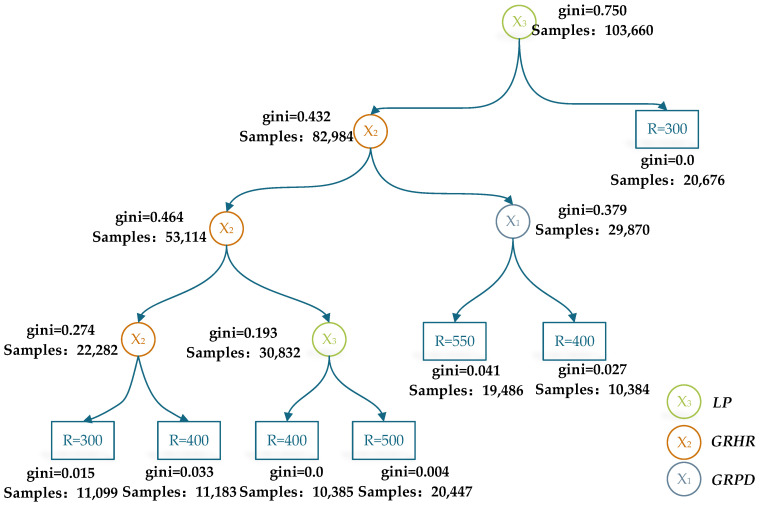
RTs model of multifeatures over horizontal curve sections.

As shown in Figure 4, the sensitivity of the *LP* was significantly larger than those of the *GRHR* and *GRPD*. To quantitate the sensitivity, the weights ($w_{i}$) of the SDW at different stages were proposed in this study, which were defined by Equation (9).

(9) $$w_{i}=\underset{m}{\sum }\frac{n_{0}g_{0}-n_{1}g_{1}-n_{2}g_{2}}{T}$$

where $n_{0}$ and $g_{0}$ represent the samples and Gini coefficient of the parent node. $n_{1}$ and $n_{2}$ indicate the samples of subnodes. $g_{1}$ and $g_{2}$ indicate the Gini coefficient of the subnodes. T is the total number of samples. m is the number of nodes. The cases with a sum of $w_{i}$ less than 1 were scaled up until the results equaled 1. After calculation, the evaluation model of driving workload was established by Equation (10) in the horizontal curve sections.

(10) $$DW=0.117DW_{\mathrm{VP}}+0.336DW_{C}+0.547DW_{M}$$

**Figure 5:**
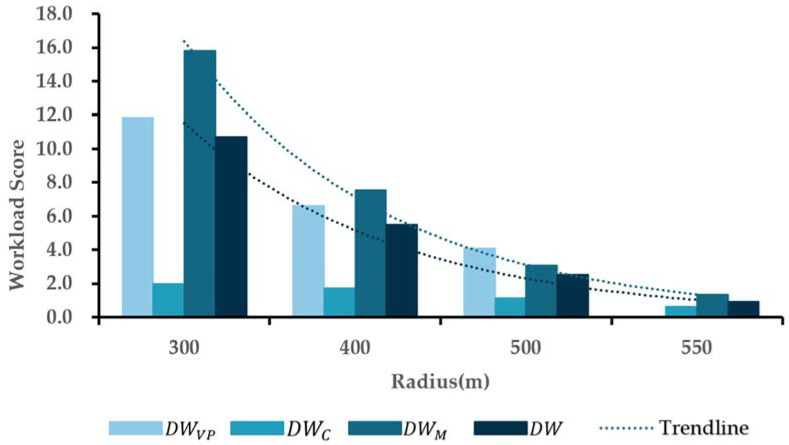
Driver workload regularity of each stage over information processing. Note: the standard section (straight-line section) is defined as 0.

It can be determined from Equation (9) that the weights of the SDW were significantly different in each stage, where $w_{\mathrm{VP}}\;$= 0.117, $w_{C}\;$= 0.336, and $w_{M}\;$= 0.547. The *DW* mainly concentrated on the maneuver stage. The reason may be that drivers generally underestimate the *OW* during the maneuver stage ($SW_{M}<OW_{M}$). The *DW*s in different horizontal curve sections were calculated by Equation (10), which were illustrated in Figure 5.

Figure 5 demonstrates that the *DW* dropped significantly with increasing radius, and the variation range decreased with increasing radius. Where the radius is 550 m, the SDW of the visual–perceptual stage was equal to that of the straight-line section ($DW_{\mathrm{VP}}=0$). The results of $DW_{\mathrm{VP}}$ indicated that a radius of more than 550 m did not influence the driver’s visual perception. However, the variation range of the SDW in the cognitive stage was lower than that in the visual–perceptual stage. The reason mainly concentrated on drivers’ subjective scores, which showed high accuracy in mental activities. Therefore, the overall difference between the *SW* and *OW* in the cognitive stage was smaller. There was a similar decrease between the *DW* and $DW_{M}$ with increasing radius. Compared to the whole processing, the *DW* of the maneuver stage represented a higher drop. It is most sensitive to the radius. Naturally, the driving performance can be considered the key indicator affected by the radius.

An increase in the radius on the horizontal curve sections led to an increase in the driving workload. This would clearly increase the difference between the subjective workload and objective workload. Compared to a radius of more than 550 m, a smaller radius may have decreased the participants’ judging accuracy, leading to a higher difference between the *SW* and *OW*. However, when the radius was equal to 550 m, there was no difference between the curve section and straight-line section in the *DW* or $DW_{\mathrm{VP}}$. Therefore, the radius of 550 m can be considered the critical radius affecting the driving workload of car drivers, which is basically consistent with the hypothesis of this study.

## 4. Discussion

In this study, a simulator was used to analyze the impact of radius on driving workload. In addition, the NASA-TLX score, *GRPD*, *GRHR*, and *LP* were combined to determine the *DW*. The results showed that the main source of *DW* caused by the radius was the maneuver stage, and its influence degree was more than 50%. A large radius (R ≥ 550 m) could result in a *DW* that was not substantially different from that produced by the straight-line section, which represented a smaller *SW* and *OW*. The results confirm the hypothesis of this study; that is, drivers are in the best *DW* standard on the straight-line section, and they have perfect performance and little mental pressure [61].

Obviously, a decrease in the radius on horizontal curve sections will affect drivers. In terms of the visual–perceptual stage, the standard deviation of the *GRPD* increased with a decrease in the radius. Because a smaller radius results in a narrower visual zone, the drivers need a longer time to search for and collect information [27,28]. The mental demand also increased as the radius decreased, and the variation was much greater than that of the *GRPD*. This process increased the difference between the *SW* and *OW*, and the driving workload also increased [57].

The analysis results of the cognitive stage showed a similar regularity with the visual–perceptual stage. Clearly, an increase in the radius could lead to a decrease in the *LP* and *GRHR*, as well as in the SDW of the cognitive and maneuver stages, which is consistent with the research results of Zheng [62]. However, the difference was greater in the maneuver stage, and the *OW* of the participants was greater than the *SW*. This indicated that the drivers underestimated the actual cost of the maneuver stage, possibly because the single act of vehicle control is often considered easier than mental activity, which leads to the underestimation of lane control workload [23,24].

The simulation results of this study especially indicated that the evaluation method for *DW* based on the multifeature combination, including *GRHR*, *GRPD*, and *LP*, significantly outperforms the method based on the above indicators alone or pairwise, which is consistent with the findings of Zheng Ling [60]. The sensitivity difference in indicators can be explained by the human model of information processing [54] in that complex driving processing results in competition for attention resources, thus allowing drivers to mobilize resources at different stages to deal with sudden competition. Of course, the different indicators explain the change in attention resources (*OW*) at different stages. This result shows that the definition of *DW* proposed in this study can be used to evaluate driving workload more accurately.

Regarding the resources demanded by driving workload, when the radius is 550 m or less, the *DW* for the maneuver stage was greater than those for the cognitive stage and visual–perceptual stage, where the $w_{i}$ of the SDW is 0.547, 0.336, and 0.117. The main reason was that the sensitivity of the indicators devoted to the SDW was related to a difference in the radius. This is consistent with the study by Waard [39]. His study also showed that the maximum lateral position is positively correlated with the rate of curvature change. This indicates that the horizontal curve sections have higher requirements for vehicle control, and drivers need to spend more attention resources on acceleration, deceleration, steering, and other operations.

It should be noted that these findings apply specifically to free driving with a speed limit of 80 km/h. The results may vary for other speed limits. Charlton [63] found that the attention resources of drivers increase at a sharp curve, and higher speeds during curve sections have higher attention resource (*OW*) demands. In addition, since the radius of the horizontal curve sections was noncontinuous, it is impossible to determine the specific threshold of the total radius of the curve sections. At the same time, physiological measurements have their own limitations, and it is difficult to obtain 100% ECG or eye-movement measurements. Factors other than radius can influence driving workload, including social attributes, personality characteristics, road structure, turn mode, and weather conditions. Therefore, it is suggested that the following studies take the above factors into account to comprehensively analyze the workload and verify the research results in field tests.

## 5. Conclusions

This paper quantifies the influence of various radii on driving workload. According to the HMP, the definition and evaluation method of *DW* is proposed in this study. The key strategy is that the NASA-TLX scores were used to express *SW*, and *OW* was represented by a combination of ECG, eye-movement, and driving performance. The regression tree model was used to determine the weight of SDW. Thus, the evaluation model of driving workload on the horizontal curve section was established. The main findings are indicated as follows:

(1) The evaluation method for *DW* based on the multifeature combination, including *GRHR*, *GRPD*, and *LP*, significantly outperforms the method based on the above indicators alone or pairwise, indicating that the definition and evaluation method based on HMP could describe driving workload more accurately in this study.

(2) Driving workload is negatively correlated with radius, and when the radius is 550 m or less, the greater the workload for drivers. This result has been confirmed in many studies. However, this study determines *DW* for various radii, where the *DW* is 0.9 with a radius of 550 m, close to that in the straight-line section. This indicates that a radius of 550 m is the critical radius affecting the driving workload of drivers.

(3) Driving workload occurs mainly in the maneuver stage, where the weight is 0.547. This result indicates that the task complexity of the horizontal curve sections mainly lies in vehicle control. In particular, drivers often underestimate the driving workload at this stage. To avoid excessive driving workload on the drivers affecting driving performance, the radius on the horizontal curve sections should not be less than 500 m, and the relevant signs should aid the guidance of vehicle control in terms of speed and lane-keeping.

(4) The subjective workload with a radius of 300 m is approximately twice that with a radius of 550 m, indicating that the influence of radius on drivers’ subjective workload is mainly concentrated in the curved sections with a small radius of less than 400 m.

(5) Compared to *SW*, *OW* is more affected by radius. Especially for the maneuver stage, the workload with a radius of 300 m is approximately three times that with a radius of 550 m, and the lateral position standard deviation is also larger. This indicates that a smaller radius causes a greater objective workload and poor driving performance, which is difficult to regulate.

Clearly, the evaluation model and definition in this study can assess driving workload more comprehensively and locate the key stages that cause driving workload to increase. This method may provide a new vision for the follow-up study of workload quantification in the human factors field. In addition, the modeling method quantified driving workload using multiple indicators, helping to provide a basis for constructing drivers’ electronic health records. This approach may provide a new perspective on methods for building intelligence systems for digital health. However, due to the limitations of experimental conditions, it is difficult to simultaneously analyze the influence of population characteristics, road environment, and climate type on driving workload. This could be another direction for future research.

## Figures and Tables

**Table 1:** Design index of the simulation model over a horizontal curve section.

Radius (m)	Average Gradient (%)	Curve Length (m)	Straight Line Length (m)	Section Length (m)	Curve Type	Turing Mode
∞	1.00	0	1280	1280	Straight-Line	-
300	1.00	775	500	1275	Basic Curve	Turn Right
400	1.00	778	500	1278	Basic Curve	Turn Right
500	1.00	774	500	1274	Basic Curve	Turn Right
550	1.00	776	500	1276	Basic Curve	Turn Right

Note: basic curve is the combination of straight-line–clothoid–circular curve–clothoid–straight-line.

**Table 2:** Pearson correlation coefficient of *OW* indicators.

Variable	*SWA*	*LP*	*GRHR*	*RMSSD*	*GRPD*	*GRBF*
*SWA*	1					
*LP*	0.078	1				
*GRHR*	−0.372 *	0.004	1			
*RMSSD*	−0.596 **	−0.554 **	0.566 *	1		
*GRPD*	−0.083	0.086	0.030	0.030	1	
*GRBF*	−0.206	−0.415 *	−0.033	0.368 *	−0.248	1

Note: * correlation is significant at the 5% level; ** correlation is significant at the 1% level.

**Table 3:** Comparison statistical test results of *DW* indicators.

Variable	Point	M–W Test		K–S Test		Variable	Point	M–W Test		K–S Test	
		*p*	Result	*p*	Result			*p*	Result	*p*	Result
MD	ZH	0.578	Accept	0.410	Accept	*LP*	ZH	0.509	Accept	0.291	Accept
	HY	0.667	Accept	0.935	Accept		HY	0.352	Accept	0.678	Accept
	QZ	0.428	Accept	0.269	Accept		QZ	0.914	Accept	0.317	Accept
	YH	0.101	Accept	0.729	Accept		YH	0.636	Accept	0.407	Accept
	HZ	0.257	Accept	0.355	Accept		HZ	0.747	Accept	0.569	Accept
TD	ZH	0.700	Accept	0.889	Accept	*GRHR*	ZH	0.732	Accept	0.767	Accept
	HY	0.481	Accept	0.194	Accept		HY	0.348	Accept	0.749	Accept
	QZ	0.224	Accept	0.812	Accept		QZ	0.541	Accept	0.885	Accept
	YH	0.151	Accept	0.854	Accept		YH	0.262	Accept	0.897	Accept
	HZ	0.801	Accept	0.799	Accept		HZ	0.866	Accept	0.292	Accept
PD	ZH	0.992	Accept	0.870	Accept	*GRPD*	ZH	0.603	Accept	0.670	Accept
	HY	0.182	Accept	0.513	Accept		HY	0.956	Accept	0.561	Accept
	QZ	0.589	Accept	0.769	Accept		QZ	0.568	Accept	0.795	Accept
	YH	0.984	Accept	0.645	Accept		YH	0.507	Accept	0.481	Accept
	HZ	0.460	Accept	0.894	Accept		HZ	0.710	Accept	0.657	Accept

Note: ZH is the point where the straight line intersects the transition curve; HY is the point where the transition curve intersects the circular curve; QZ is the middle point of the circular curve; YH is the point where the circular curve intersects the transition curve; HZ is the point where the transition curve intersects the straight line.

**Table 4:** Subjective workload over different radii.

Horizontal Curve (m)	300	400	500	550	∞
$SW_{\mathrm{VP}}$	21.800	16.200	13.800	9.600	6.800
$SW_{C}$	21.600	18.600	14.400	8.400	6.200
$SW_{M}$	26.000	21.400	15.800	13.400	8.000

**Table 5:** Linear influence factor over different radii.

Horizontal Curve (m)	300	400	500	550	∞
$LIF_{\mathrm{VP}j}$	1.086	1.045	1.061	1.046	0.742
$LIF_{Cj}$	1.121	0.963	0.757	0.443	0.354
$LIF_{Mj}$	3.915	2.150	1.861	1.380	0.749

**Table 6:** Objective workload over different radii.

Horizontal Curve (m)	300	400	500	550	∞
$OW_{\mathrm{VP}}$	9.953	9.580	9.727	9.587	6.800
$OW_{C}$	19.634	16.867	13.251	7.766	6.200
$OW_{M}$	41.820	28.964	18.876	14.738	8.000

**Table 7:** SDW over different radii.

Horizontal Curve (m)	300	400	500	550
$DW_{\mathrm{VP}}$	11.847	6.620	4.073	0.013
$DW_{C}$	1.197	1.733	1.149	0.634
$DW_{M}$	15.820	7.564	3.076	1.338

**Table 8:** Accuracy of three classification algorithms under different feature inputs.

Data Set	Algorithm	*GRHR*	*GRPD*	*LP*	*GRHR + GRPD*	*GRHR + LP*	*GRPD + LP*	*GRHR + GRPD + LP*
Training Set	ANNs	0.585	0.257	0.542	0.654	0.686	0.618	0.783
	SVM	0.719	0.468	0.856	0.892	0.879	0.889	0.904
	RTs	0.491	0.213	782	0.881	0.912	0.946	0.974
Test Set	ANNs	0.468	0.217	0.359	0.488	0.642	0.578	0.747
	SVM	0.522	0.313	0.511	0.727	0.814	0.838	0.863
	RTs	0.417	0.201	0.642	0.763	0.858	0.937	0.948

Note: the accuracy is the average value of 10-fold cross-validation.

## Data Availability

The data are available upon request.

## Abbreviations

HMP	the Human Model of Information Processing
*DW*	Driving Workload
SDW	Driving Workload of Each Stage (visual–perceptual, cognition, maneuver)
*OW*	Objective Workload
*SW*	Subjective Workload
ECG	Electrocardiograph
*LP*	Lateral Position
*PD*	the Pupil Diameter
*BF*	the Blink Frequency
*HR*	the Heart Rate
*GRPD*	the Growth Rate of Pupil Diameter
*GRHR*	the Growth Rate of Heart Rate
*GRBF*	the Growth Rate of Blink Frequency
*SWA*	the Steering Wheel Angle
*RMSSD*	the Root Mean Square of Successive Differences between Adjacent RR Intervals
HRV	the Heart Rate Variability
MD	Mental Demand
PD	Physical Demand
TD	Temporal Demand
VP	Visual–Perceptual Stage
M	Maneuver Stage
C	Cognition Stage
ANNs	the Artificial Neural Networks
SVM	the Support Vector Machines
RTs	the Regression Trees
*LIF*	the Linear Influence Factor
